# The influence of *Aloe vera* with mesenchymal stem cells from dental pulp on bone regeneration: characterization and treatment of non-critical defects of the tibia in rats

**DOI:** 10.1590/1678-7757-2018-0103

**Published:** 2019-04-11

**Authors:** Isadora Mello Vilarinho SOARES, Gustavo Vicentis de Oliveira FERNANDES, Larissa Cordeiro CAVALCANTE, Yulla Klinger Pereira de Carvalho LEITE, Dayseanny de Oliveira BEZERRA, Maria Acelina Martins de CARVALHO, Carmen Milena Rodrigues Siqueira CARVALHO

**Affiliations:** 1 Universidade Federal do Piauí, Departamento de Patologia e Prática Odontológica, Teresina, Piauí, Brasil.; 2 Universidade Católica de Portugal, Departamento de Periodontia, Viseu, Portugal.; 3 Universidade Federal do Piauí, Departamento de Microfisiologia Veterinária, Teresina, Piauí, Brasil.

**Keywords:** Bone regeneration, Stem cells, Stem cell transplantation, Osteopontin, Herbal medicines

## Abstract

**Objective:**

This study aimed to evaluate the inflammatory effect and bone formation in sterile surgical failures after implantation of a collagen sponge with mesenchymal stem cells from human dental pulp (hDPSCs) and *Aloe vera*.

**Material and Methods:**

*Rattus norvegicus* (n=75) were divided into five experimental groups according to treatment: G1) control (blood clot); G2) Hemospon^®^; G3) Hemospon^®^ in a culture medium enriched with 8% *Aloe vera*; G4) Hemospon^®^ in a culture medium containing hDPSCs and G5) Hemospon^®^ in a culture medium enriched with 8% *Aloe vera* and hDPSCs. On days 7, 15 and 30, the animals were euthanized, and the tibia was dissected for histological, immunohistochemistry and immunofluorescence analyses. The results were analyzed using nonparametric Kruskal-Wallis test and Dunn’s post-test.

**Results:**

On days 7 and 15, the groups with *Aloe vera* had less average acute inflammatory infiltrate compared to the control group and the group with Hemospon^®^ (p<0.05). No statistically significant difference was found between the groups regarding bone formation at the three experimental points in time. Osteopontin expression corroborated the intensity of bone formation. Fluorescence microscopy revealed positive labeling with Q-Tracker^®^ in hDPSCs before transplantation and tissue repair.

**Conclusion:**

The results suggest that the combination of Hemospon^®^, *Aloe vera* and hDPSCs is a form of clinical treatment for the repair of non-critical bone defects that reduces the inflammatory cascade’s effects.

## Introduction

Bone defects are one of the most dangerous problems found in patients seeking oral rehabilitation. These defects cause difficulty for treating and restoring the patient’s smile and functionality, especially if the defect is critical. Many studies involving bone repair were undertaken to gather new knowledge, techniques and materials to solve the problem.^1^ ^-^ ^3^ Among these, stem cell research has grown exponentially in recent years due to the recognition that this therapy has great potential in regenerative medicine.^4^

The repair of bone defects is characterized by three overlapping phases: the formation of blood clots, bone formation, and bone remodeling. During the second phase, osteoprogenitor cells migrate to the wound site, proliferate and differentiate into osteoblasts that segregate local growth factors, the extracellular matrix and induce mineralization. Therefore, the tissue’s regeneration depends on the availability of these precursor cells and on the presence of stimuli required to recruit and stimulate these cells.^1^ ^,^ ^3^

Thus, the tissue’s regeneration can be improved if the material has osteoinductive characteristics. With this purpose, studies involving stem cells have grown exponentially due to the recognition that this therapy has great potential in regenerative medicine.^4^

Dental pulp stem cells (DPSCs) are a vital source of osteoprogenitor cells that have a fundamental role in the mechanisms related to the pulp tissue’s plasticity. They are also involved in inducing tissue regeneration based on their differentiation ability in culture and expression of mesenchymal stem cell (MSC) markers.^5^ ^-^ ^7^ These characteristics were observed for the dental pulp of permanent teeth (hDPSCs)^5^ ^,^ ^6^ and exfoliated deciduous teeth (SHED)^7^ in humans.

The pursuit of care and improvements in the tissue repair process and in the pharmacodynamic anti-inflammatory and immunoregulatory properties of natural products have drawn attention, and many of them were tested on tissues. The plant species of the *Aloe* (*Liliaceae*) genus were extensively studied due to their medicinal properties. Among them is *Aloe vera*, a species that contains many biologically active substances^8^ that are able to provide significant anti-inflammatory, antibacterial, hypoglycemic, immunomodulating, wound healing and regenerative properties.^9^ Additionally, acemannan, a polysaccharide extracted from *Aloe vera*, is a biomolecule with potential for tissue regeneration, playing a significant role in cell proliferation, extracellular matrix synthesis and mineralization.^1^ ^,^ ^10^ ^-^ ^12^

Therefore, an important question became the focus of this work: could *Aloe vera* enhance and accelerate bone repair? This study aimed to evaluate bone repair in non-critical defects of rat tibias after implantation of a collagen sponge (Hemospon^®^) colonized with mesenchymal stem cells from human dental pulp (hDPSCs) and *Aloe vera*.

## Material and methods

This study was approved by the Ethics Committee on Human Research (0218.0.045.000-11) and by the Ethics Committee on Animal Experiments (066/14), both from the Federal University of Piauí (Brazil). Seventy-five male *Rattus norvegicus* rats were used. The animals were divided into five experimental groups according to the materials tested: 1. control group (blood clot); 2. Hemospon^®^; 3. Hemospon^®^ in a culture enriched with 8% *Aloe vera*; 4. Hemospon^®^ in a culture containing mesenchymal stem cells from human dental pulp (hDPSCs); and 5. Hemospon^®^ in a culture enriched with 8% of *Aloe vera* and hDPSCs. The hDPSCs used for transplant were in their fifth passage. Each group was evaluated at three experimental points in time: after 7, 15 and 30 days.

### Cellular preparation

#### Isolation and expansion of hDPSCs

Two intact third molars indicated for extraction were collected from the patient after he had studied and signed the informed consent form (Index 1). After extraction, the teeth were cleaned with 70% alcohol (Adv Farma, Nova Odessa, SP, Brazil). A tear was made on each tooth at the height of the cementoenamel junction with a sterilized spherical diamond tip No. 1014 (KG Sorensen, Cotia, SP, Brazil) with 0.9% saline irrigation (Adv Farma, Nova Odessa, SP, Brazil). Using No. 222 forceps (Golgran, São Caetano do Sul, São Paulo, Brazil), the tooth was fractured at the point of the tear and the dental pulp was removed with a Hedstrom 40 file (Dentsply Maillefer, Ballaigues, Switzerland). The tissue was stored in a falcon tube containing 2 ml sterile phosphate buffered saline (PBS 0.01 M, pH=7.4), and immediately transferred to the Morphophysiology Laboratory at the University of Piauí (Brazil).

The collected material was washed three times in PBS supplemented with 10% penicillin-streptomycin (100 U/ml penicillin, 100 μg/ml streptomycin). The pulp tissue was dissociated mechanically with a sterile No. 24 scalpel blade (Brasmed, Paulínia, SP, Brazil) on a 35-mm petri dish. The dish contained a “complete basal” culture medium D-MEM/F-12 (GIBCO Life Technologies, São Paulo, SP, Brazil), supplemented with 20% fetal bovine serum (GIBCO Life Technologies, São Paulo, SP, Brazil), 1% penicillin-streptomycin and 1% L-glutamine, after which it transferred to six-well plates. Subsequently, the material was incubated in an incubator (Thermo Fisher Scientific, Waltham, Massachusetts, USA) with 5% CO_2_ atmosphere at 37°C. The culture was monitored for the evaluation of cell growth, and the culture medium was modified for three days.

When they reached 80% confluence, the cultured cells were subcultured, avoiding the induction of cell differentiation by contact. The culture was subjected to trypsinization with 1 mL of trypsin-EDTA and incubated for 5 minutes. The addition of 2 ml of the culture medium deactivated trypsin. The mixture was added to a 15-ml conical tube and centrifuged at 20°C at 1500 rpm for 10 minutes. The supernatant was discarded. The pellet obtained was re-suspended in 3 ml of culture medium and transferred to a 25-cm^2^ culture flask.

The cultures were expanded in 25 cm^2^ bottles until the fourth passage, replicated with twice the original area to reach 80% confluence, and cryopreserved. Each culture was washed with PBS, subjected to an enzymatic dissociation process using trypsin-EDTA digestion, and re-suspended in a freezing medium (40% FBS, 50% DMEM low glucose and 10% dimethylsulfoxide, Sigma-Aldrich, St. Louis, Missouri, USA). After this process, the cells were transferred to freezing tubes (cryo-tubes, TPP) in 1.0x10^6^ cells/ml and kept at -196°C in liquid nitrogen.

As the specific isolation of stem cells was not performed, the cell culture used in the present study represents a mixed culture of pulp cells with the presence of a subpopulation of stem cells.

#### Kinetics and cell viability

A growth curve was created for kinetic analysis in duplicate after ten days and the cell yield was estimated in 24-hour intervals. Cells at the fifth passage were seeded on six-well plates at a concentration of 1x10^4^ cells in 1 ml of medium for each well. For cell viability observation and to calculate the concentration, a 10-μL cell suspension aliquot and 10 μL of Blue Trypan (GIBCO Life Technologies, São Paulo, SP, Brazil) were homogenized. 10 μL of the mixture was transferred to a hemocytometer, and the number of cells was counted in an optical microscope (10x objective). Cell concentration was determined by counting the cells in four diagonal fields, the total number of living cells being multiplied by the dilution factor and 10^4^ (depth of the camera and correction *per* ml) by dividing this value by four. To determine the viability of the samples, the dead cells stained with Trypan Blue (GIBCO, Life Technologies, São Paulo, SP, Brazil) were counted, and the percentage of living cells was calculated using the total number of cells counted.

#### Differentiation assays

Differentiation assays were performed to break down the hDPSCs into adipocytes, osteocytes and chondrocytes, using a medium supplemented with specific differentiation-inducing factors. For adipogenic differentiation, 2x10^4^ cells were cultured on a 24-well plate and after reaching 80% confluence. The medium was removed and replaced using adipogenic induction with the StemPro Adipogenesis Differentiation Kit (GIBCO Life Technologies, São Paulo, SP, Brazil). The differentiation’s evolution was observed using an inverted microscope. To observe the deposition of fat after 14 days in the culture, the differentiated cells were stained using Oil Red.

For osteogenic differentiation, 6x10^4^ cells were cultured on a 24-well plate. After 48 hours, the cells were stimulated for 21 days with an osteogenesis differentiation medium StemPro Differentiation Kit (GIBCO Life Technologies, São Paulo, SP, Brazil). For three days, the culture medium was changed. The deposition of calcium was observed by staining them with Alizarin Red.

For chondrogenic differentiation, 3x10^5^ cells were cultured with a DMEM/F-12 medium and 20% fetal bovine serum on a 96-well plate. After 48 hours, spheroidal bodies spontaneously formed in the culture on the wells, and the medium was replaced with a differentiation medium of the StemPro Chondrogenesis Differentiation Kit (GIBCO Life Technologies, São Paulo, SP, Brazil). It was changed twice a week for 21 days. Spheroids bodies were subjected to routine laboratory histology and stained with an Alcian Blue solution.

#### Flow cytometry

The characterization of the hDPSCs using flow cytometry was based on the expression analysis of cell surface molecules CD14 (Anti-CD14 FITC Alexa Fluor 488 - Thermo Fisher Scientific, Waltham, Massachusetts, USA), CD45 (Anti-Alexa Fluor 488 - Thermo Fisher Scientific, Waltham, Massachusetts, USA) CD105 (anti-CD105 Alexa Fluor 488 - Thermo Fisher Scientific, Waltham, Massachusetts, USA), yielding 30,000 events *per* tested sample.

Sixth-passage cells were used, thawed, cultured and trypsinized to reach 80% to 90% confluence in the 25-cm^2^ flask. After centrifugation at 462 g for 10 minutes, the cells were counted in a Neubauer chamber and centrifuged again at 462 g for 10 minutes. The pellet was re-suspended in 250 µL of PBS divided into five aliquots with 50 µL each, in 15 ml Falcon tubes. The four antibodies were each placed in a tube (CD14 – 10 µL, CD45 – 10 µL, CD105 – 5 µL). One tube was used as the control. The tubes were left in an oven at 37°C with 5% CO_2_ and 95% humidity for 30 minutes without light. After this period, two centrifugations at 462 g were performed for 5 minutes. 400 µL of PBS was added to the resulting pellet. After this, they were transferred to a cytometer tube, where data acquisition was conducted followed by analysis in a flow cytometer. The results were plotted in a histogram for evaluation.

#### Kinetics and cell viability in the presence of Aloe vera

To evaluate the kinetics and cellular viability in a medium supplemented with *Aloe vera*, we repeated the same method. The same cell population in the same passage were used. This time, we used the D-MEM/F-12 culture medium supplemented with 20% fetal bovine serum (GIBCO Life Technologies, São Paulo, SP, Brazil), 1% penicillin-streptomycin, 1% L-glutamine and 8% *Aloe Vera* extract.^1^ ^,^ ^11^ ^,^ ^12^

The *Aloe vera* extract was obtained from the plant leaf (*Aloe barbadensis* Miller) provided by the Center for Research on Medicinal Plants at the Health Sciences Center, UFPI. To obtain the extract, the sheets were cut 5 cm from the stalk, washed in tap water and placed in a container with distilled water for 24 hours to avoid contamination of the gel with the sap at the time of extraction. After 24 hours, the sheets were again washed in tap water, and the gel was removed by gently scraping the parenchyma with a 24 spatula. Under laminar flow, the gel was filtered using a 25-mm PureFlo filter disc with 5 μm micron (ZenPure Corporation, São Paulo, SP, Brazil) and stored in a falcon tube without exposure to light.

#### Labeling with Qtracker®

To verify that the transplanted cells remained on the defect site and contributed to bone consolidation, the cells were labeled with Qdots^®^, 655 nm emission, 450-615 nm excitation (Qtracker^®^ Cell Labeling Kit, GIBCO Life Technologies, São Paulo, SP, Brazil). The labeling is provided by the presence of highly fluorescent nanocrystals in the cells’ cytoplasm. To confirm the label, 10 μL of the cells subjected to labeling were placed under a histological slide and coverslip and examined with a BX41 fluorescence microscope (Olympus, Shinjuku, Tokyo, Japan).

#### Scaffold

Hemospon^®^ fragments, a lyophilized and hydrolyzed collagen sponge with porcine origin (Technew, Rio de Janeiro, RJ, Brazil), which had a size that was compatible with the size of the bone defect (2 mm/2 mm), were obtained. The fragments remained immersed in 2 mL of the culture medium in an incubator with 5% CO_2_ at 37°C, for 24 hours before the surgical procedures. For groups 4 and 5, we used a culture medium containing 10^6^ cells/mL.

## Surgical procedures

The animals were premedicated with 0.2% acepromazine intramuscularly (IM) in 5 mg/kg doses. The anesthetic combination was created using 2% Xylazine Hydrochloride (Anasedan^®^, Vetbrands, Vinhedo, SP, Brazil) IM in 5 mg/kg doses, combined with ketamine (Dopalen^®^, Vetbrands, Vinhedo, SP, Brazil) IM in 100 mg/kg doses.

After this, trichotomy was performed on the right hindlimb using a polvidine-iodine topical antiseptic (Riodeine^®^, Rioquímica, São José do Rio Preto, SP, Brazil) while isolating the tibia with sterile surgical drapes. Surgical access to the right tibia of each animal was performed with a linear 20 mm incision in the craniocaudal direction, using a No. 24 scalpel blade (Brasmed, Paulínia, SP, Brazil). After that, the dissection of skin, muscle, and periosteum was performed to expose the bone surface.

Using copious irrigation with 0.9% saline solution (Adv Farma, Nova Odessa, SP, Brazil) and a No. 4 round bur with a long steel rod (JET, Morrisburg, Ontario, Canada) mounted on a surgical handpiece (KaVo, Joinville, SC, Brazil), a bone defect was made on the tibia with approximate 2 mm diameter and a depth that reached the spinal canal. The defect was filled according to experimental group, after which the suture of the muscle was sewn with chromic absorbable catgut using needle No. 4.0 (PolySuture^®^, São Sebastião do Paraiso, MG, Brazil), and the skin was sewn with a surgical silk thread also using needle No. 4.0 (Shalon^®^, Goiânia, GO, Brazil).

The animals were monitored during the postoperative period and were given paracetamol (Tylenol^®^, São Paulo, SP, Brazil) diluted in drinking water, administered orally in 2 mg/ml doses for 48 hours. They received antibiotics intramuscularly (PPU Pencivet^®^ Plus, Intervet, São Paulo, SP, Brazil) in 1 ml/kg body weight doses immediately after surgery to prevent postoperative infections. On postoperative days 7, 15 and 30, five animals from each group were euthanized by overdosing them with 1 g of a sodium thiopental anesthetic, administered intraperitoneally (25 mg/kg) (Thiopentax^®^, Cristália, São Paulo, SP, Brazil).

## Processing and histological analysis

Each animal had its right tibia dissected, and the parts were fixed in 10% neutral buffered formalin for 48 hours. After setting, the specimens were decalcified in a hydrochloric acid solution (5% per hour; 10% for 30 minutes and 15% for 15 minutes). The fragments were dehydrated in solutions with increasing ethanol concentrations, cleared in xylene and fixed in histological resin. The specimens were embedded in paraffin after longitudinal 3 μm sections were made. The sections were stained with hematoxylin and eosin (HE) for histomorphological analysis and the identification of areas of new bone formation and inflammation.

**Figure 1:**
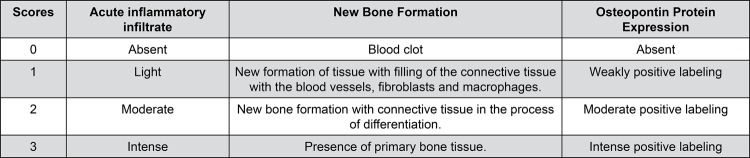
Scores assigned to histopathologic events for the semi-quantitative evaluation of the acute inflammatory infiltrate, new bone formation and osteopontin expression

**Figure 2:**
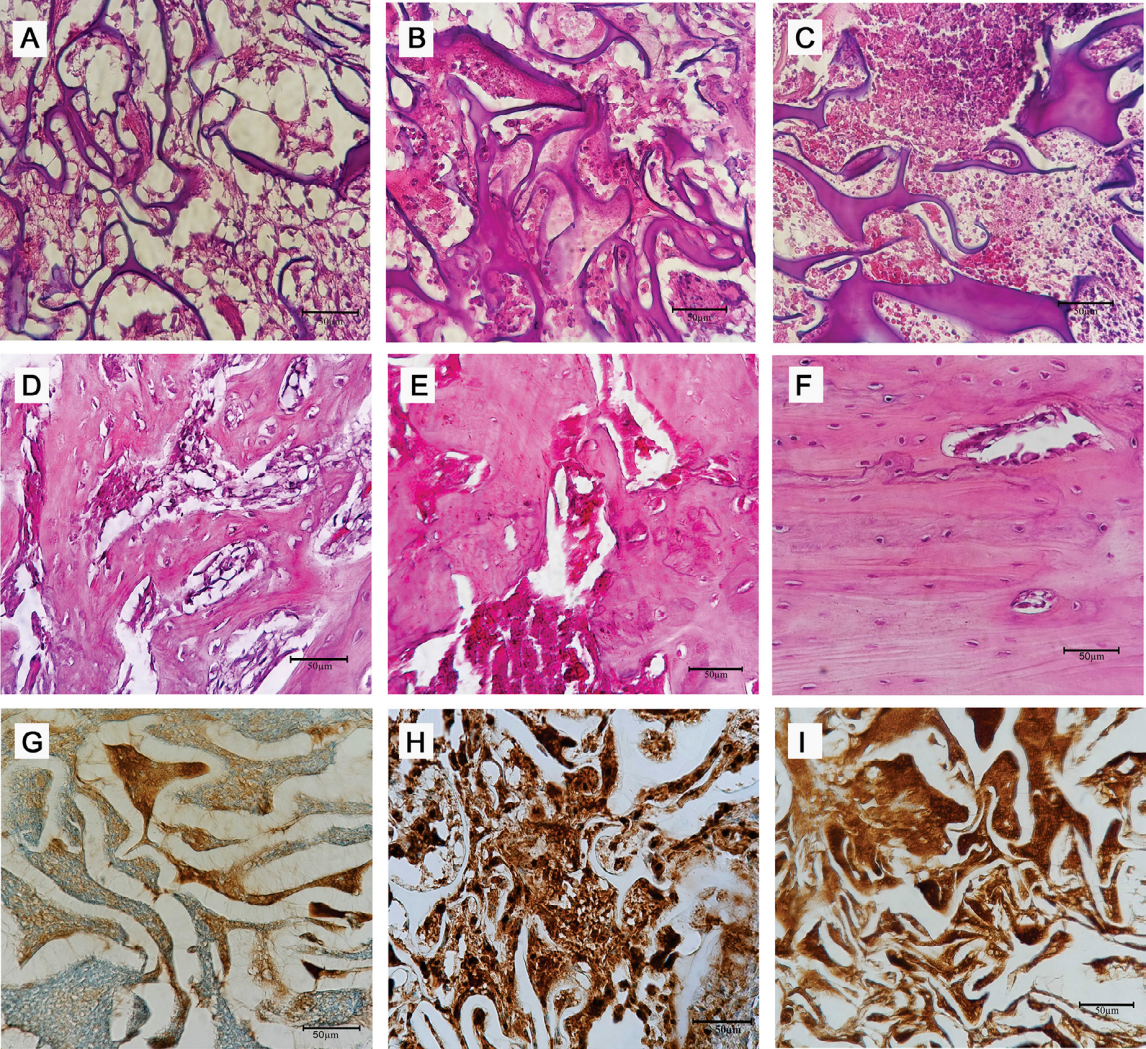
Photomicrographs of bone defects produced on the right tibia of animals, exemplifying the histopathologic patterns used for the allocation of scores in the semi-quantitative analysis of the acute inflammatory infiltrate (a) score 1 (b) score 2 and (c) score 3; of bone formation: (d) score 1 (e) score 2, and (f) score 3 and of osteopontin expression: (g) score 1 (h) score 2 and (i) score 3. Bar: 50 μm

The sections were examined under a binocular light microscope (NIKON, Eclipse E600, Tokyo, Japan). The descriptive (qualitative) and semi-quantitative analyses of the blades were conducted by a blinded medical pathologist, considering the acute inflammatory infiltrate and new bone formation parameters. The observed phenomena and the criteria used to measure them semi-quantitatively, by assigning scores, are shown in Figures 1 and 2 (A-F).^13^

## Immunohistochemistry

For the immunohistochemical reactions, primary monoclonal antibodies were (Santa Cruz Biotechnology, Dallas, Texas, USA, sc-21742 reference titer 1:200) developed against osteopontin (OPN). Tissue samples were embedded in paraffin, cut into longitudinal 3 mm sections and placed on silanized slides. The sections were deparaffinized in an oven at 60°C for one hour. The slides were then submitted to the Staining Platform IHC/ISH (BenckMark GX Ventana, Mannheim, Germany) under the following protocol: deparaffinization, CC1 for 60 min, antibody for 32 min, amplifier, hematoxylin for 8 min and bluing for 4 min. Soon after being washed, the slides were cleaned with mild soap and running water followed by three washings in absolute alcohol and two in xylene, positioning the coverslip and fixing it with Entellan (Merck Millipore, Darmstadt, Germany).

The immunohistochemical evaluation of the OPN expression was performed in a semi-quantitative manner according to the intensity of the chromogen substance’s impregnation, with the assignment of scores (Figure 1). The immunoreactivity was found in the bone matrix component of the regenerated tissues (Figure 2, G-I).^14^ Human kidney tissue was used as an external positive control. As a negative control, the sections were submitted to the same procedure, with omission of the antibody.

To obtain intra-examiner agreement, two readings were conducted using 10% of the cuts at different times (kappa=0.875).

## Fluorescence microscopy

Tissue samples already embedded in paraffin were cut into longitudinal 6 µm sections and placed on slides before being analyzed under the light. The fluorescence microscopy analysis was performed using a BX41 fluorescence microscope (Olympus, Shinjuku, Tokyo, Japan).

## Statistical analysis

Data were tabulated using the SPSS software version 21.0 for Windows. The data obtained were subjected to a normality test (Kolmogorov-Smirnov) and homogeneity of variance (Levene). The data on cell behavior in the presence of *Aloe vera* were regular and homogeneously distributed, being subsequently subjected to parametric analysis (ANOVA). Nonparametric Kruskal-Wallis and Dunn’s *post-*test were used to compare the histological and immunohistochemical results due to the non-normal distribution of the data. The tests were performed with 5% statistical significance level (p<0.05).

## Results

### Cell behavior

**Figure 3:**
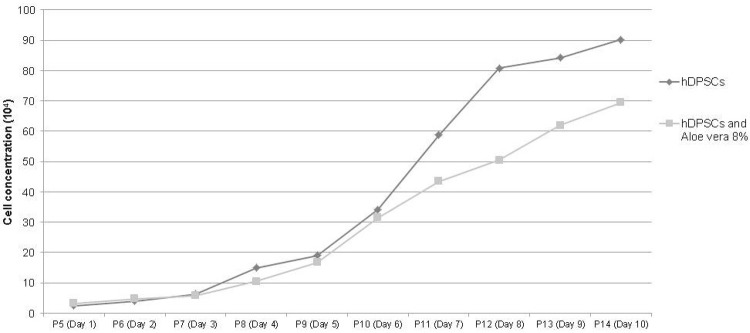
Comparison between mean cell concentration in the growth of the hDPSCs’ curve using standard culture medium and medium supplemented with 8% *Aloe vera* for 10 days

Around the 7^th^ day of cultivation, the first adherent cells released by the explant tissue were observed, showing a rounded form that gradually lengthened, acquired a fibroblastoid format, and reached 80% confluence after 15 days in the culture medium. The study of the growth curve of the mesenchymal stem cells in fifth passage examined the proliferative behavior of the cells in the culture, with maximum cell concentration occurring on the 10^th^ day of culture. The hDPSCs demonstrated high potential for replication and multiplication in the presence or absence of 8% *Aloe vera*, with no statistically significant difference in the analyzed days (p=0.485) (Figure 3). The viability of the hDPSCs cultivated in contact with *Aloe vera* was shown to be higher than 90% during the 10 days of culture.

**Figure 4:**
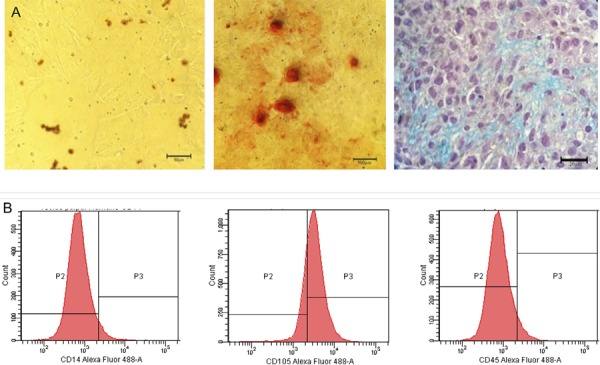
(A) From left to right, adipogenic differentiation stained with Oil Red showing lipid droplets, 50 μm bar, osteogenic differentiation stained with Alizarin Red showing calcium deposits and matrix, 100 μm bar, and chondrogenic differentiation stained with Alcian Blue showing glycosaminoglycans, 20 μm bar. (B) Cytometry flow of fibroblast cells originated from human permanent tooth pulp tissue, with expression of CD105 and non-expression of CD14 and CD45

The hDPSCs demonstrated the potential of multi-lineage differentiation after being induced in a specific medium *in vitro*. Figure 4A shows the adipogenic, osteogenic and chondrogenic differentiation from left to right. Regarding the phenotypic profile of the hDPSCs using flow cytometry, the results showed that approximately 60% of the cells expressed CD105, a mesenchymal cell indicator. In relation to CD14 and CD45, no marking was found as expected, excluding the possibility that these are hematopoietic cells (Figure 4B).

### Histological and immunohistochemical analysis

**Figure 5:**
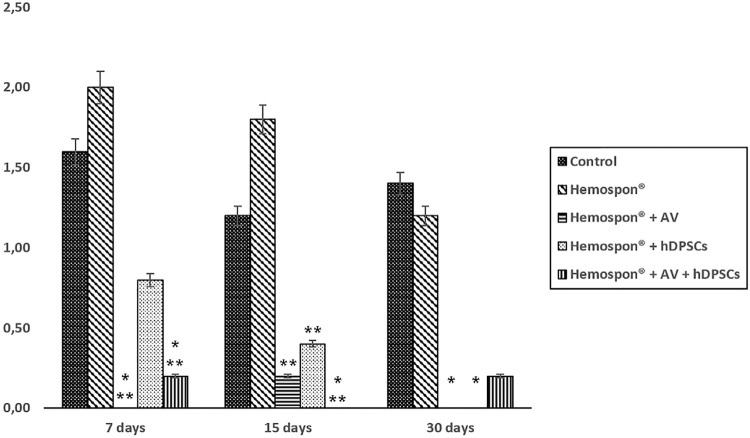
Average scores for the experimental groups’ acute inflammatory infiltrate at each evaluated time

The acute inflammatory infiltrates decreased as time passed for the Hemospon^®^ group and the Hemospon^®^ and hDPSCs groups. After 7 and 15 days, the *Aloe vera* groups had a lower average with statistically significant difference (p<0.05) between the control and Hemospon^®^ groups. After 30 days, lower averages were found for the groups that had Hemospon^®^ and *Aloe vera*, Hemospon^®^ and hDPSCs, and Hemospon^®^, hDPSCs, and *Aloe vera*, with statistically significant differences between the first two compared to the control group (p<0.05) (Figure 5).

* Significant difference in relation to the control group; ** Significant difference in relation to the Hemospon^®^ group

**Figure 6:**
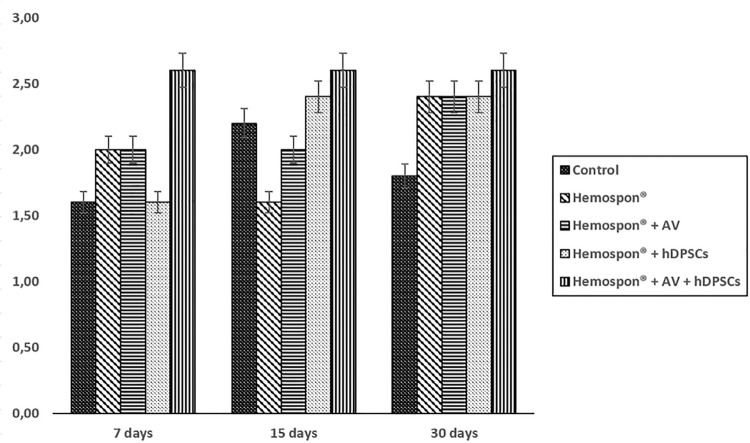
Average scores for the experimental groups’ bone formation in each evaluated time

**Figure 7:**
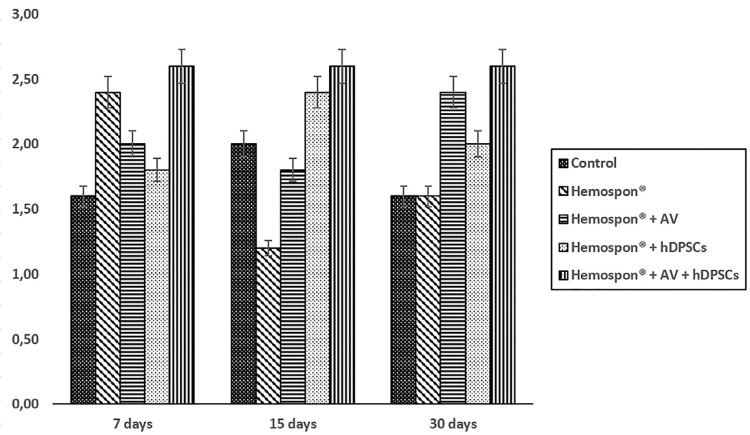
Average scores for the experimental groups’ osteopontin expression in each evaluated time

During the seven-day period, the group with Hemospon^®^, hDPSCs, and *Aloe vera* featured repair, based on the presence of primary bone tissue. The bone formation of other groups was shown to be moderate, with young connective tissue in process of differentiation after 7 and 15 days (Figure 6). Corroborating this, the OPN expression was at the same degree of new bone formation during the three experimental periods (Figure 7).

### Fluorescence microscopy

**Figure 8:**
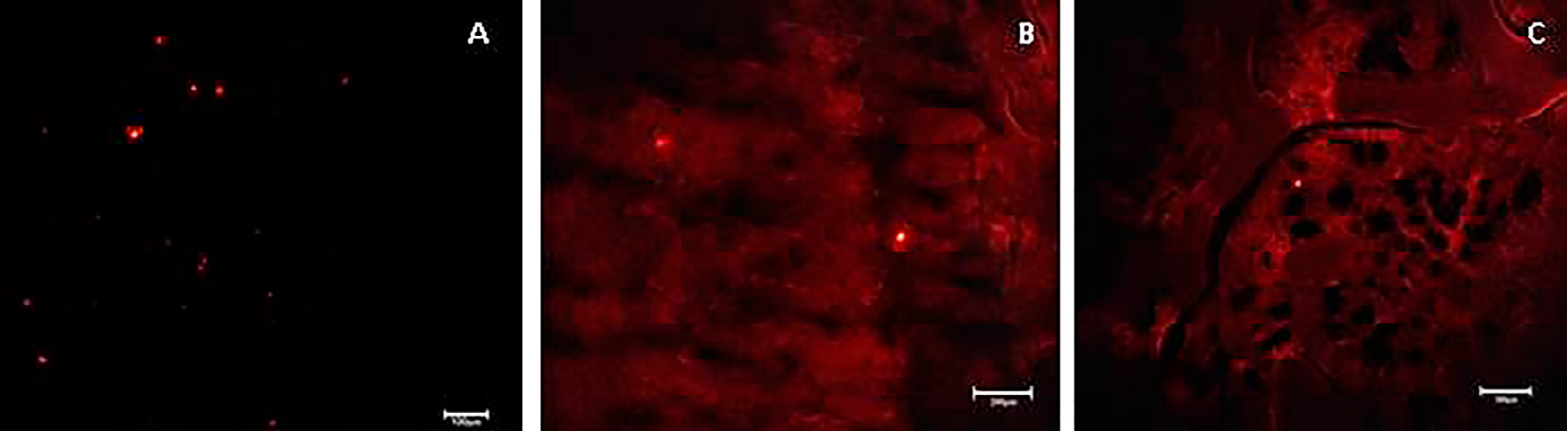
Fluorescence microscopy image, showing (A) positive staining of the hDPSCs using Q-Tracker®, 100 μm bar; (B) the presence of cells with positive staining using Q-tracker® in regenerated tissue 30 days after the *in vivo* transplantation in the group treated with Hemospon® and hDPSCs, 200 μm bar, and (C) in the group treated with Hemospon®, hDPSCs and *Aloe vera*, after 7 days, 200 μm bar

The fluorescence microscopy analysis revealed positive labeling for Q-Tracker^®^ in the hDPSCs before transplantation (Figure 8A) and in fixed tissue (Figures 8B e 8C).

## Discussion

The potential for differentiation, self-renewal and proliferation of stem cells (SCs) led to the development of studies involving tissue repair and remodeling, even though the plasticity of these cells can be greater and differentiate them into specific cells according to the tissue of origin.^15^ In our study, the behavior of the hDPSCs revealed the expansion of capacity and differentiation in three cell lines: adipogenic, osteogenic and chondrogenic (Figure 3),^5^ ^-^ ^7^ ^,^ ^16^ according to the basic requirements for the identification of SCs.^17^

When testing the immunophenotype, there was no labeling of CD14 and CD45, but the results were positive for CD105, stressing the importance of compliance with the steps of characterization of the hDPSCs. This process confirms that the stem cell is indeed a mesenchymal stem cell (MSC), according to the International Society for Cellular Therapy. The population of MSCs must express CD105, CD73 and/or CD90, and should have negative results for CD45, CD34, CD14 or CD11b, CD79a or CD19 and HLA class II.^17^ ^,^ ^18^ This can be measured with flow cytometry, like in our study, to help confirm the growth of mesenchymal stem cells and exclude the possibility of hematopoietic cell populations.

In the presence of a medium enriched with 8% *Aloe vera*, the hDPSCs continued to experience exponential growth during ten days of cultivation, like the control group. In both groups, the formation of colonies and of a fibroblast morphology that is characteristic of MSCs was observed.^5^ ^,^ ^7^ Furthermore, the results demonstrated that the component was not toxic to the cell culture since there was no greater number of dead cells observed during the viability analysis.

SCs in bone reconstitute the presence of a biocompatible, resorbable, radiolucent, and porous support material with osteoconductive capability that is necessary.^19^ Collagen sponges are currently used in various therapeutic practices due to their high availability, secure purification, biocompatibility and non-toxicity.^20^ Additionally, their use as a “scaffold” in bone defects has shown positive results whether they are colonized with SCs or not.^21^ In the study, we opted for a Hemospon^®^ collagen sponge (Technew, Rio de Janeiro, RJ, Brazil), a dental material for clinical use, which is easy to access and has a web of collagen fibers that allows the adhesion of cells. It is also capable of being reabsorbed within four weeks.^22^ This short period could be a disadvantage for the treatment of critical bone defects, but it would be a good alternative to accelerate the repair process in non-critical defects. The results obtained in this study allow us to suggest that Hemospon^®^ can be used as a “scaffold” for SCs in tissue repair.

The *Aloe vera* was used in the study as an anti-inflammatory factor to aid in bone repair.^11^ ^,^ ^23^ ^,^ ^24^ Statistically significant (p<0.05) inflammatory infiltration results were found in our research, where the groups treated with the herbal medicine showed better results. The reduction of inflammation may be explained by the anti-inflammatory properties of *Aloe vera*, due to the immunomodulatory effect of acetylated polysaccharides mannose, acemannan and veracilglucanos B and C, which regulate the expression of inflammatory mediators such as interleukins 6 and 8.^23^ Similar results were observed in the treatment of bone defects using Mineral Trioxide Aggregate and *Aloe vera*, with significant reduction of the inflammatory cascade’s effects.^24^

The treatment of bone defects with MSCs showed that in the presence of a favorable mechanical and biological environment, there is proliferation and differentiation of these cells in osteoblasts, and that therapy with SC decreases the defect’s healing time.^21^ In our study, there was no statistically significant difference between the groups in relation to bone formation. However, in the group treated with Hemospon^®^, *Aloe vera*, and hDPSCs, greater new bone formation rate was observed, associated with mild inflammation in the initial 7-day trial period. It was suggested that in addition to its anti-inflammatory properties, *Aloe vera* also positively influences bone formation in the presence of hDPSCs.

The herbal medicine’s repair activity was partially attributed to the presence of mannose-6-phosphate polysaccharide, which acts as an immunostimulant that activates macrophages, increases cytokine release, and stimulates an increase in the replication of fibroblasts which are partially responsible for tissue repair.^25^ Studies have demonstrated the positive effect of acemannan, the main polysaccharide extracted from *Aloe vera*, in the expression of growth factors,^9^ stimulation of regeneration of bone, cementum and periodontal ligament^12^ and induction of bone formation, proliferation, and differentiation of the osteoblast of SCs.^1^ Shanmugavel, et al.^26^ (2014) demonstrated that polycaprolactone/*Aloe vera*/Silk fibroin/Hydroxyapatite nanofibrous scaffolds with stem cells have appropriate physical-chemical and biological properties that can be used as biomimetic scaffolds for bone tissue regeneration.

In the immunohistochemical analysis of the samples, the antibody developed against OPN was chosen, since this is one of the main non-collagen proteins involved in osteogenesis and in the bone remodeling process.^27^ Studies on the temporal OPN expression during bone formation *in vitro* and *in vivo* revealed a biphasic pattern where the OPN is produced early in the differentiation of bone cells with high levels expressed after mineralization begins. High levels of expression continued in association with bone remodeling. This protein is an indicator of the early bone formation stages.^28^ These observations are consistent with the results found in this study, where OPN expression was present after seven days, indicating that these groups already had proliferation and osteoblastic activity.

For a complete evaluation of bone defect repair mediated using SCs, it is necessary to make sure that the transplanted cells remained in the defected area and contributed to bone healing.^29^ The culture of cells with fluorescent markers is an easy method that is commonly used to track cells *in vivo* and for monitoring them.^30^ Once inside the cells, Qdots^®^ provide a stable and robust fluorescence that can be traced back for several generations while not being transferred to adjacent cells in a population. In our study, the labeling of cells before transplantation with Qdots^®^ and in regenerated tissue in the groups treated with hDPSCs in the presence or absence of *Aloe vera* was confirmed, indicating that bone regeneration was guided by the hDPSCs (Figure 8).

## Conclusions

These results suggest that Hemospon^®^, *Aloe vera*, and hDPSCs are a promising clinical procedure to repair non-critical defects and speed up the repair process, reducing the inflammatory cascade’s effects.

## References

[1] Boonyagul S, Banlunara W, Sangvanich P, Thunyakitpisal P (2014). Effect of acemannan, an extracted polysaccharide from Aloe vera, on BMSCs proliferation, differentiation, extracellular matrix synthesis, mineralization, and bone formation in a tooth extraction model. Odontology.

[2] Costa NM, Yassuda DH, Sader MS, Fernandes GV, Soares GD, Granjeiro JM (2016). Osteogenic effect of tricalcium phosphate substituted by magnesium associated with Genderm® membrane in rat calvarial defect model. Mater Sci Eng C Mater Biol Appl.

[3] Dimitriou R, Jones E, McGonagle D, Giannoudis PV (2011). Bone regeneration: current concepts and future directions. BMC Med.

[4] Desiderio V, Tirino V, Papaccio G, Paino F (2014). Bone defects: molecular and cellular therapeutic targets. Int J Biochem Cell Biol.

[5] Gronthos S, Brahim J, Li W, Fisher LW, Cherman N, Boyde A (2002). Stem cell properties of human dental pulp stem cells. J Dent Res.

[6] Gronthos S, Mankani M, Brahim J, Robey PG, Shi S (2000). Postnatal human dental pulp stem cells (DPSCs) in vitro and in vivo. Proc Natl Acad Sci U S A.

[7] Miura M, Gronthos S, Zhao M, Lu B, Fisher LW, Robey PG (2003). SHED: stem cells from human exfoliated deciduous teeth. Proc Natl Acad Sci U S A.

[8] Bernardes I, Felipe Rodrigues MP, Bacelli GK, Munin E, Alves LP, Costa MS (2012). Aloe vera extract reduces both growth and germ tube formation by Candida albicans. Mycoses.

[9] Bhat G, Kudva P, Dodwad V (2011). Aloe vera: Nature's soothing healer to periodontal disease. J Indian Soc Periodontol.

[10] Chantarawaratit P, Sangvanich P, Banlunara W, Soontornvipart K, Thunyakitpisal P (2014). Acemannan sponges stimulate alveolar bone, cementum and periodontal ligament regeneration in a canine class II furcation defect model. J Periodontal Res.

[11] Jettanacheawchankit S, Sasithanasate S, Sangvanich P, Banlunara W, Thunyakitpisal P (2009). Acemannan stimulates gingival fibroblast proliferation; expressions of keratinocyte growth factor-1, vascular endothelial growth factor, and type I collagen; and wound healing. J Pharmacol Sci.

[12] Jittapiromsak N, Sahawat D, Banlunara W, Sangvanich P, Thunyakitpisal P (2010). Acemannan, an extracted product from Aloe vera, stimulates dental pulp cell proliferation, differentiation, mineralization, and dentin formation. Tissue Eng Part A.

[13] Pretel H, Lizarelli RF, Ramalho LT (2007). Effect of low-level laser therapy on bone repair: histological study in rats. Lasers Surg Med.

[14] Routray S, Kheur SM, Kheur M (2013). Osteopontin: a marker for invasive oral squamous cell carcinoma but not for potentially malignant epithelial dysplasias. Ann Diagn Pathol.

[15] Monteiro BG, Serafim RC, Melo GB, Silva MC, Lizier NF, Maranduba CM (2009). Human immature dental pulp stem cells share key characteristic features with limbal stem cells. Cell Prolif.

[16] Alkaisi A, Ismail AR, Mutum SS, Ahmad ZA, Masudi S, Abd Razak NH (2013). Transplantation of human dental pulp stem cells: enhance bone consolidation in mandibular distraction osteogenesis. J Oral Maxillofac Surg.

[17] Dominici M, Le Blanc K, Mueller I, Slaper-Cortenbach I, Marini F, Krause D (2006). Minimal criteria for defining multipotent mesenchymal stromal cells. The International Society for Cellular Therapy position statement. Cytotherapy.

[18] Horwitz EM, Le Blanc K, Dominici M, Mueller I, Slaper-Cortenbach I, Marini F (2005). Clarification of the nomenclature for MSC: The International Society for Cellular Therapy position statement. Cytotherapy.

[19] Nitzsche H, Lochmann A, Metz H, Hauser A, Syrowatka F, Hempel E (2010). Fabrication and characterization of a biomimetic composite scaffold for bone defect repair. J Biomed Mater Res A.

[20] Oryan A, Alidadi S, Moshiri A, Maffulli N (2014). Bone regenerative medicine: classic options, novel strategies, and future directions. J Orthop Surg Res.

[21] Zavatti M, Bertoni L, Maraldi T, Resca E, Beretti F, Guida M (2015). Critical-size bone defect repair using amniotic fluid stem cell/collagen constructs: effect of oral ferutinin treatment in rats. Life Sci.

[22] Donzelli E, Salvadè A, Mimo P, Viganò M, Morrone M, Papagna R (2007). Mesenchymal stem cells cultured on a collagen scaffold: in vitro osteogenic differentiation. Arch Oral Biol.

[23] Devaraj A, Karpagam T (2011). Evaluation of anti-inflammatory activity and analgesic effect of Aloe vera leaf extract in rats. Int Res J Pharm.

[24] Fé JL, Coelho CA, Damascena GM, Soares IM, Alves FR, Santos IM (2014). Aloe vera as vehicle to mineral trioxide aggregate: study in bone repair. Rev Odontol UNESP.

[25] Aro AA, Nishan U, Perez MO, Rodrigues RA, Foglio MA, Carvalho JE (2012). Structural and biochemical alterations during the healing process of tendons treated with Aloe vera. Life Sci.

[26] Shanmugavel S, Reddy VJ, Ramakrishna S, Lakshmi BS, Dev VG (2014). Precipitation of hydroxyapatite on electrospun polycaprolactone/Aloe vera/silk fibroin nanofibrous scaffolds for bone tissue engineering. J Biomater Appl.

[27] Gordjestani M, Dermaut L, De Ridder L, Waele P (2007). Osteopontin and bone repair in rabbit tibial defect. Eur J Orthop Surg Traumatol.

[28] Sodek J, Chen J, Nagata T, Kasugai S, Todescan R, Li IW (1995). Regulation of osteopontin expression in osteoblasts. Ann N Y Acad Sci.

[29] Shah BS, Clark PA, Moioli EK, Stroscio MA, Mao JJ (2007). Labeling of mesenchymal stem cells by bioconjugated quantum dots. Nano Lett.

[30] Dupont KM, Sharma K, Stevens HY, Boerckel JD, García AJ, Guldberg RE (2010). Human stem cell delivery for treatment of large segmental bone defects. Proc Natl Acad Sci U S A.

